# Supplementary material for: Dimeric Transmembrane Structure of the SARS-CoV-2 E Protein
Source: Commun Biol. 2023 Nov 1;6:1109. doi: 10.1038/s42003-023-05490-x (PMC10620413; doi:10.1038/s42003-023-05490-x)
Supplement: Supplementary file 1 — Supplementary Table and Figures [file 42003_2023_5490_MOESM1_ESM.pdf]

Dimeric Transmembrane Structure of the SARS-CoV-2 E Protein

Rongfu Zhang[1,2,6], Huajun Qin[1,6], Ramesh Prasad[4], Riqiang Fu[2], Huan-Xiang Zhou[4,5]*, and Timothy A. Cross[1,2,3]*

[1]Department of Chemistry and Biochemistry, Florida State University, Tallahassee, FL 32306

[2]National High Magnetic Field Laboratory, Tallahassee, FL 32310

[3]Institute of Molecular Biophysics, Florida State University, Tallahassee, FL 32306

[4]Department of Chemistry, University of Illinois Chicago, Chicago, IL 60607

[5]Department of Physics, University of Illinois Chicago, Chicago, IL 60607

[6]Contributed equally to this work

*Correspondence to be addressed: hzhou43@uic.edu or timothyacross@gmail.com

Supplemental Table and Figures

Supplementary Table 1. PISEMA resonance assignments and those back-calculated from the refined structure.

Residue	^{15}N Anisotropic Chemical Shift (ppm)		^{1}H-^{15}N Dipolar Coupling (kHz)	
	Assigned[a]	Back-calculated[b]	Assigned[a]	Back-calculated[b]
I13	220	221 ± 5	8.9	9.2 ± 0.4
V14	223	224 ± 3	9	8.8 ± 0.5
V17	223	221 ± 5	9	9.1 ± 0.4
L18	223	225 ± 3	9	9.2 ± 0.5
L19	223	222 ± 4	9	9.5 ± 0.5
F20	220	219 ± 3	9.3	9.7 ± 0.5
L21	223	223 ± 3	9	8.9 ± 0.6
A22	224.5	228 ± 4	8.9	9.2 ± 0.5
F23	220	224 ± 3	9.3	9.6 ± 0.4
V24	223	221 ± 4	9	9.5 ± 0.5
V25	223	216 ± 7	9	9.1 ± 0.6
F26	220	224 ± 3	9.3	9.0 ± 0.6
L27	223	222 ± 3	9	9.9 ± 0.4
L28	223	223 ± 3	9	9.4 ± 0.5
V29	223	222 ± 4	9	9.1 ± 0.4
T30	222.6	222 ± 3	8.8	9.7 ± 0.4
L31	223	219 ± 4	9	9.6 ± 0.4
A32	224.5	226 ± 4	8.9	9.0 ± 0.5
I33	220	225 ± 3	8.9	8.9 ± 0.5
L34	223	219 ± 4	9	10.0 ± 0.5
T35	206.3	219 ± 4	8.6	9.2 ± 0.4
A36	215.3	223 ± 7	9.7	8.7 ± 0.5
L37	223	222 ± 4	9	9.2 ± 0.5
I13	220	221 ± 5	8.9	9.2 ± 0.4

[a]Residues of the same amino-acid type were assigned to the same peak position. Exceptions are T35 and A36.

[b]Presented as mean ± SD calculated among the 14 models.

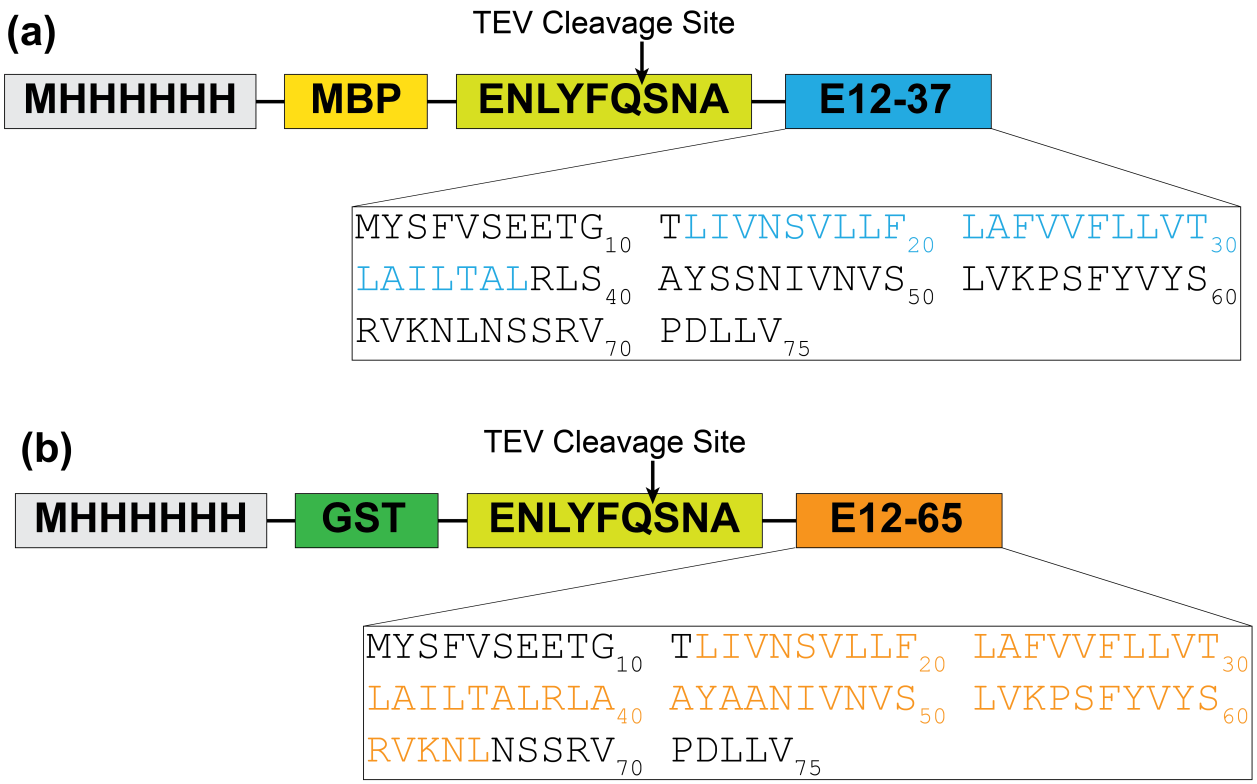

Supplementary Figure 1. Schematic of SARS-CoV-2 E protein constructs utilized for bacterial expression and purification. (a) MBP fusion construct for E_{12-37}; E_{1-52}, E_{7-43}, and E_{8-41}. (b) GST fusion construct for E_{12-65}. The TEV cleavage site within the sequence ENLYFQSNA is indicated with an arrow.

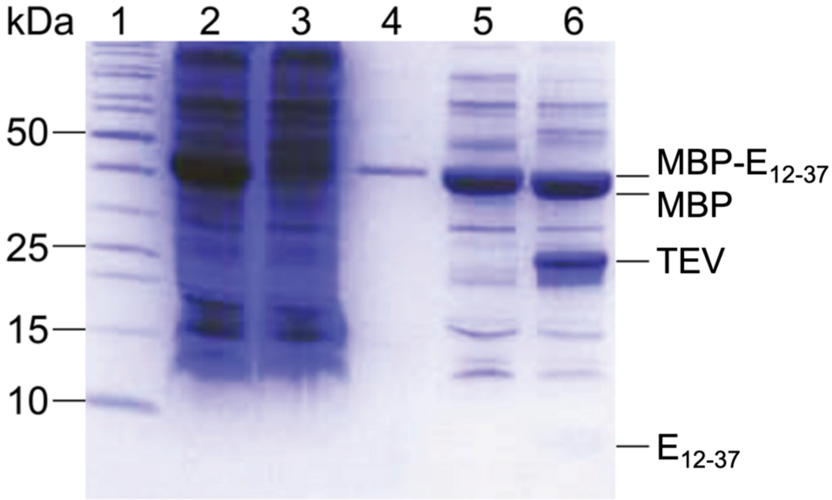

Supplementary Figure 2. SDS-PAGE gel for the $E_{12\text{-}37}$ purification. Lane 1, marker; lane 2, cell lysate; lane 3, column flow-through; lane 4, column wash; lane 5, elute of MBP-$E_{12\text{-}37}$ fusion protein; lane 6, post TEV cleavage. Protein bands of interest are indicated and labeled to the right of the gel. The band for $E_{12\text{-}37}$ is actually a dimer, which could easily be mistaken as a monomer when guided only by the high molecular weight markers.

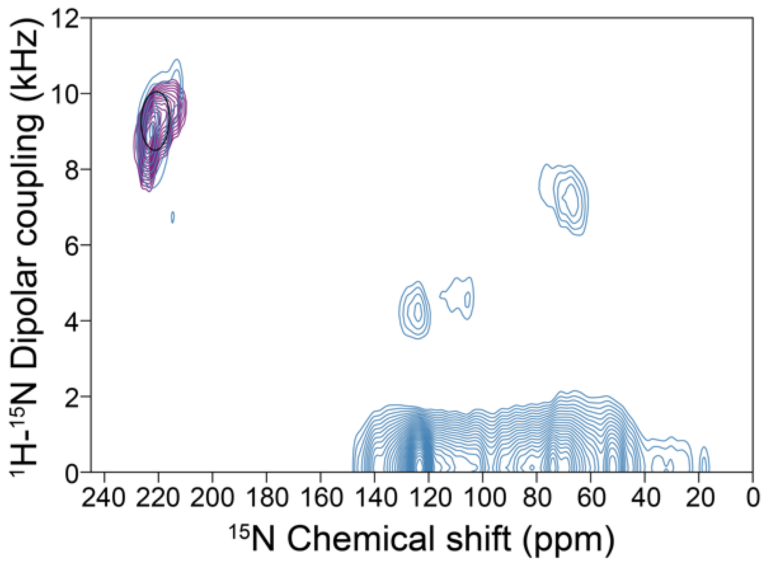

Supplementary Figure 3. Overlay of 2D PISEMA spectra of ^{15}N-Val labeled $E_{12\text{-}37}$ (purple) and ^{15}N-Val labeled $E_{8\text{-}41}$ (teal) in aligned POPC/POPG bilayers, with a PISA wheel of 6° tilt superimposed.

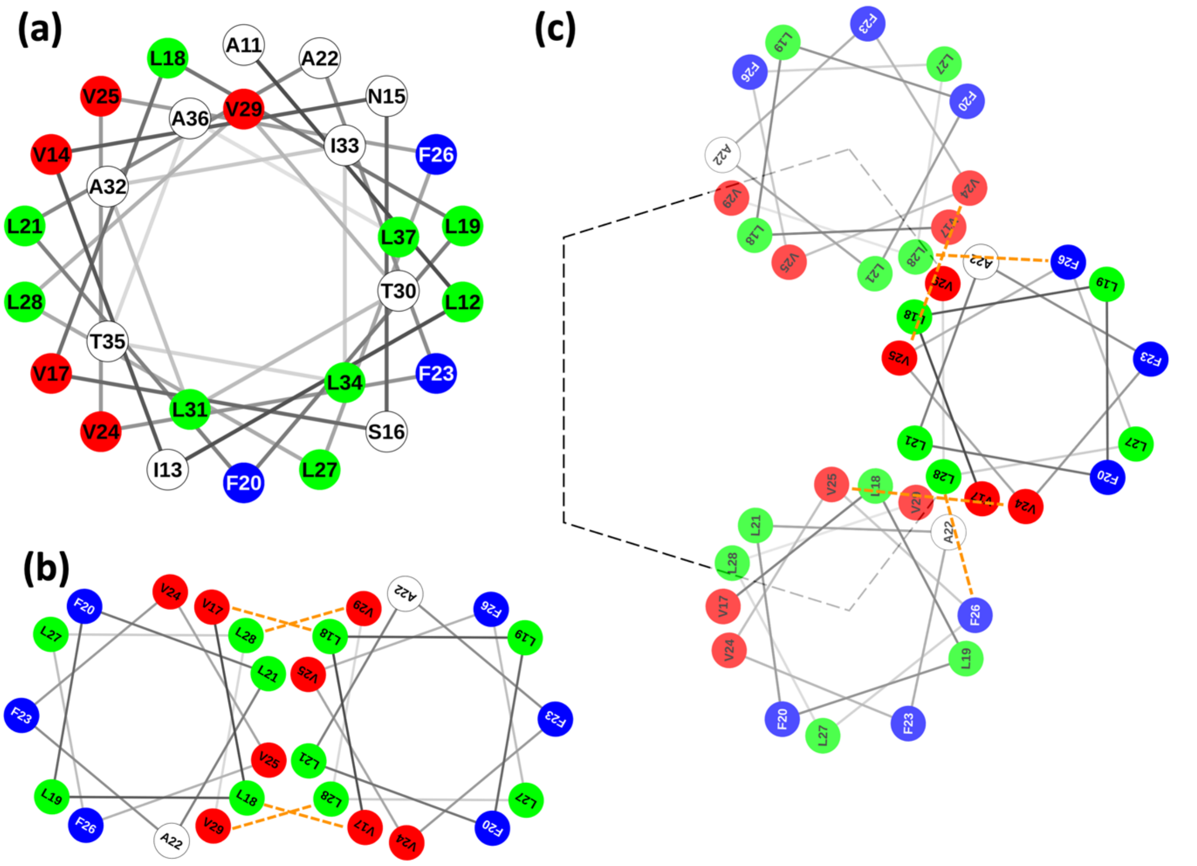

Supplementary Figure 4. Possible interhelical interfaces based on the helical wheel of $E_{12\text{-}37}$. (a) The helical wheel, emphasizing the fact that five Val residues (red) locate on one face of the helix, three Phe residues (blue) locate on the opposite face of the helix, while 9 Leu residues (green) populate the entire helical surface. (b) A symmetric dimer interface that satisfies all the dipolar restraints from MAS ssNMR, including ≤ 7 Å Cα-Cα distances for all Val17-Leu18 and Leu28-Val29 pairs (indicated by dashed lines). (c) The asymmetric interhelical interface in a pentamer, minimizing the burial of Phe residues but unable to avoid Val24-Val25 (7.0 Å) and Phe26-Leu28 (7.1 Å) Cα-Cα contacts (indicated by dashed lines).

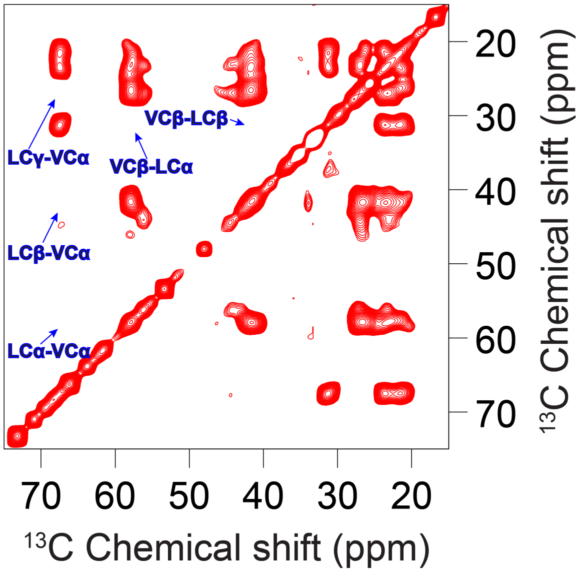

Supplementary Figure 5. ^{13}C-^{13}C correlation spectrum of an equimolar mixture of ^{13}C-Leu labeled E_{12-37} and ^{13}C-Val labeled E_{12-37} in POPC/POPG liposomes at a mixing time of 100 ms. More contour levels are shown than in Figure 4 to emphasize the absence of cross peaks between labeled sites.

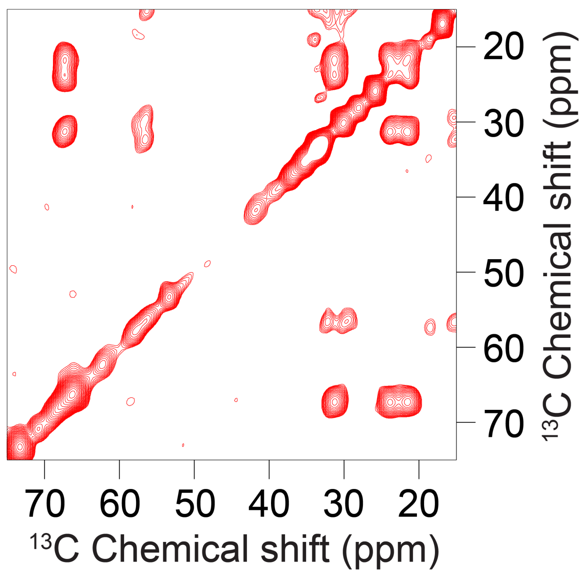

Supplementary Figure 6. ^{13}C-^{13}C correlation spectrum of an equimolar mixture of two versions of the $E_{12\text{-}37}$ mutant, one with ^{13}C-Val labeling and one with ^{13}C-Met labeling, in POPC/POPG liposomes at a mixing time of 600 ms.

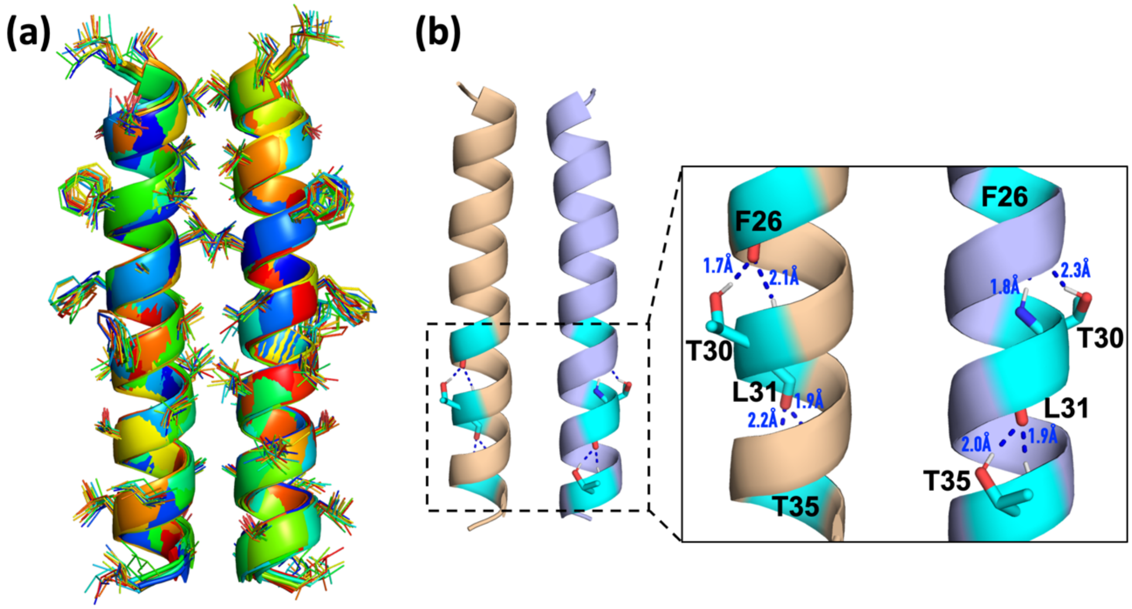

Supplementary Figure 7. Refined structure of the $E_{12\text{-}37}$ dimer. (a) Superposition of 14 models from replicate refinement simulations. (b) Sidechain-backbone hydrogen bonding of Thr30 and Thr35.

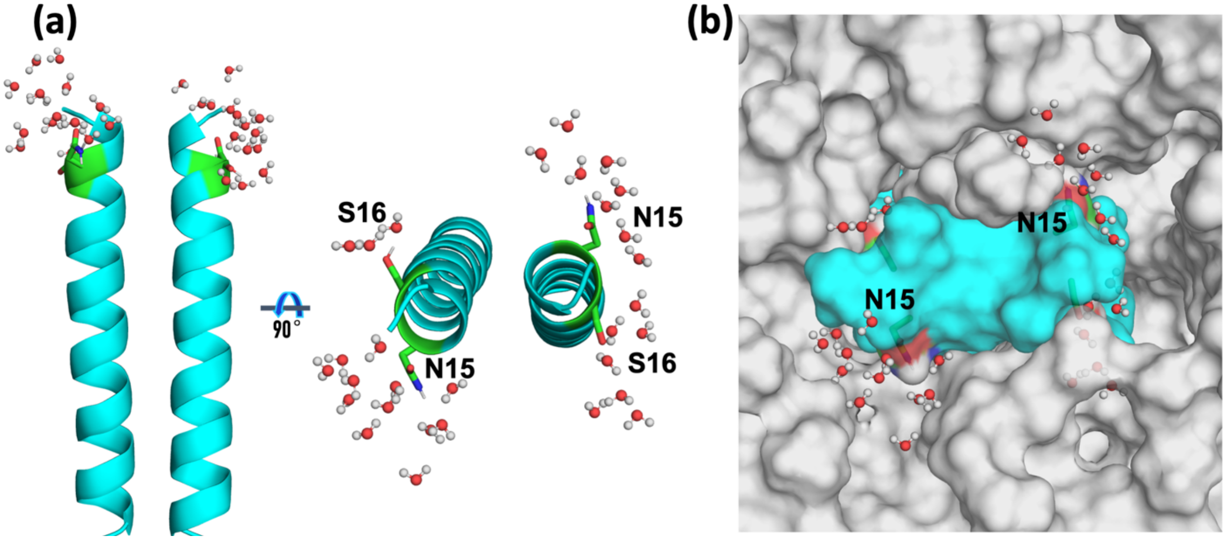

Supplementary Figure 8. Exposure of Asn15 and Ser16 sidechains to water at the membrane surface. (a) Hydrogen bonding of Asn15 and Ser16 with water molecules. (b) Water-filled pockets around Asn15, potentially serving as drug-binding sites. The membrane and $E_{12\text{-}37}$ dimer are rendered as surface in gray and cyan, respectively, except that Asn15 and Ser16 are rendered with C, O, and N atoms in green, red, and blue, respectively.

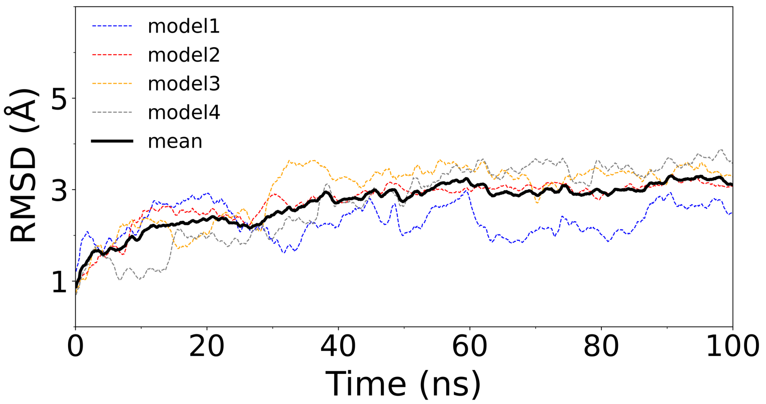

Supplementary Figure 9. Root-mean-square-deviations (RMSDs) of snapshots along 100-ns simulations, in reference to the starting structures. The simulations were started from four of the 14 ssNMR models. The steady values in the second half of the simulations indicate convergence of the simulations. Data points were calculated at 0.1 ns intervals; curves were smoothed as running averages of 11 points. These simulations were carried out to demonstrate the stability of the starting structure, and did not probe any long-time events that would require enhanced sampling.

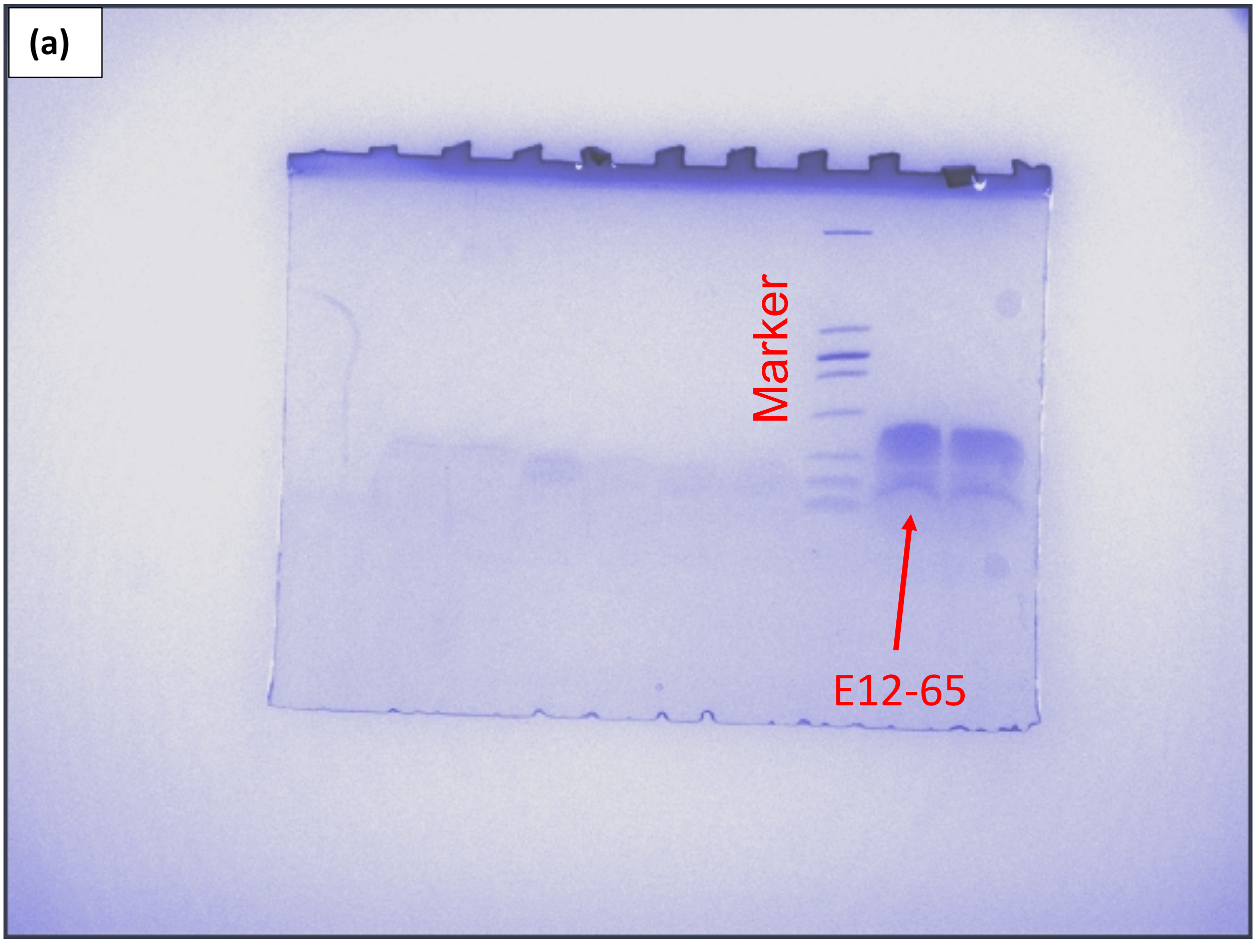

Supplementary Figure 10. Uncropped gel images. (a) E_{12-65}; cropped version shown in Figure 1a, with the aspect ratio increased by 33%.

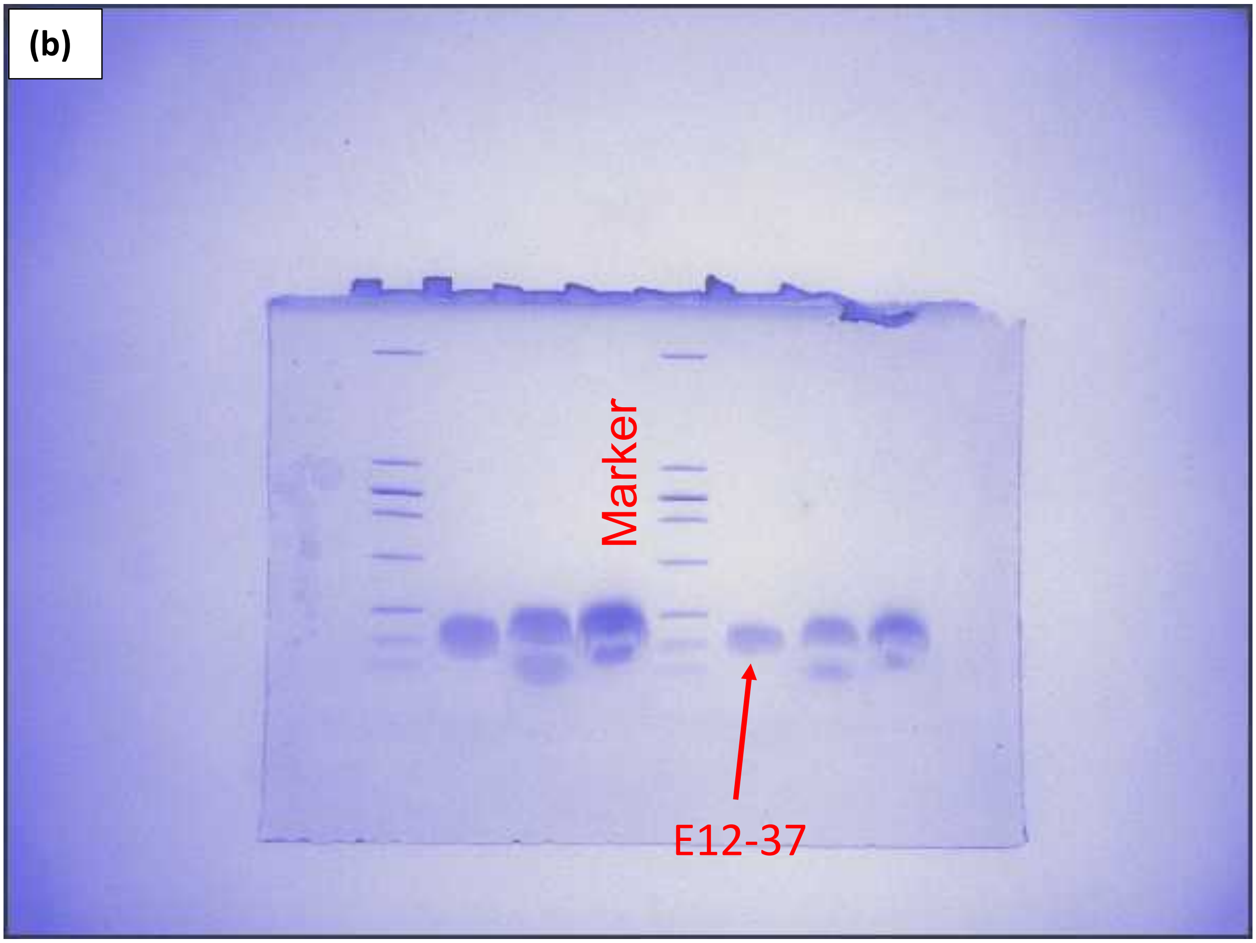

Supplementary Figure 10. Uncropped gel images. (b) $E_{12\text{-}37}$; cropped version shown in Figure 1b, with the aspect ratio increased by 13%.

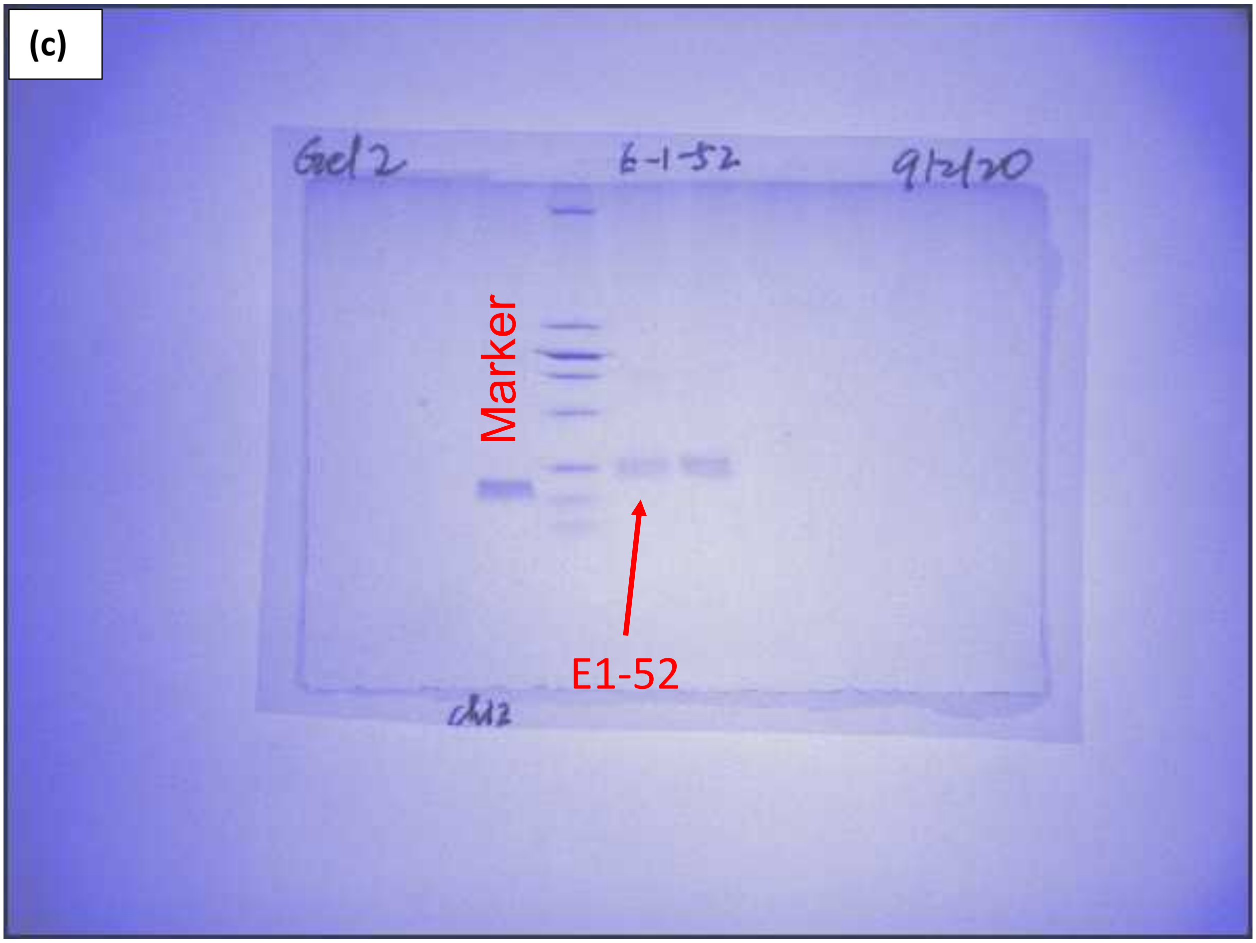

Supplementary Figure 10. Uncropped gel images. (c) E_{1-52}; cropped version shown in Figure 1c, with the aspect ratio increased by 10%.

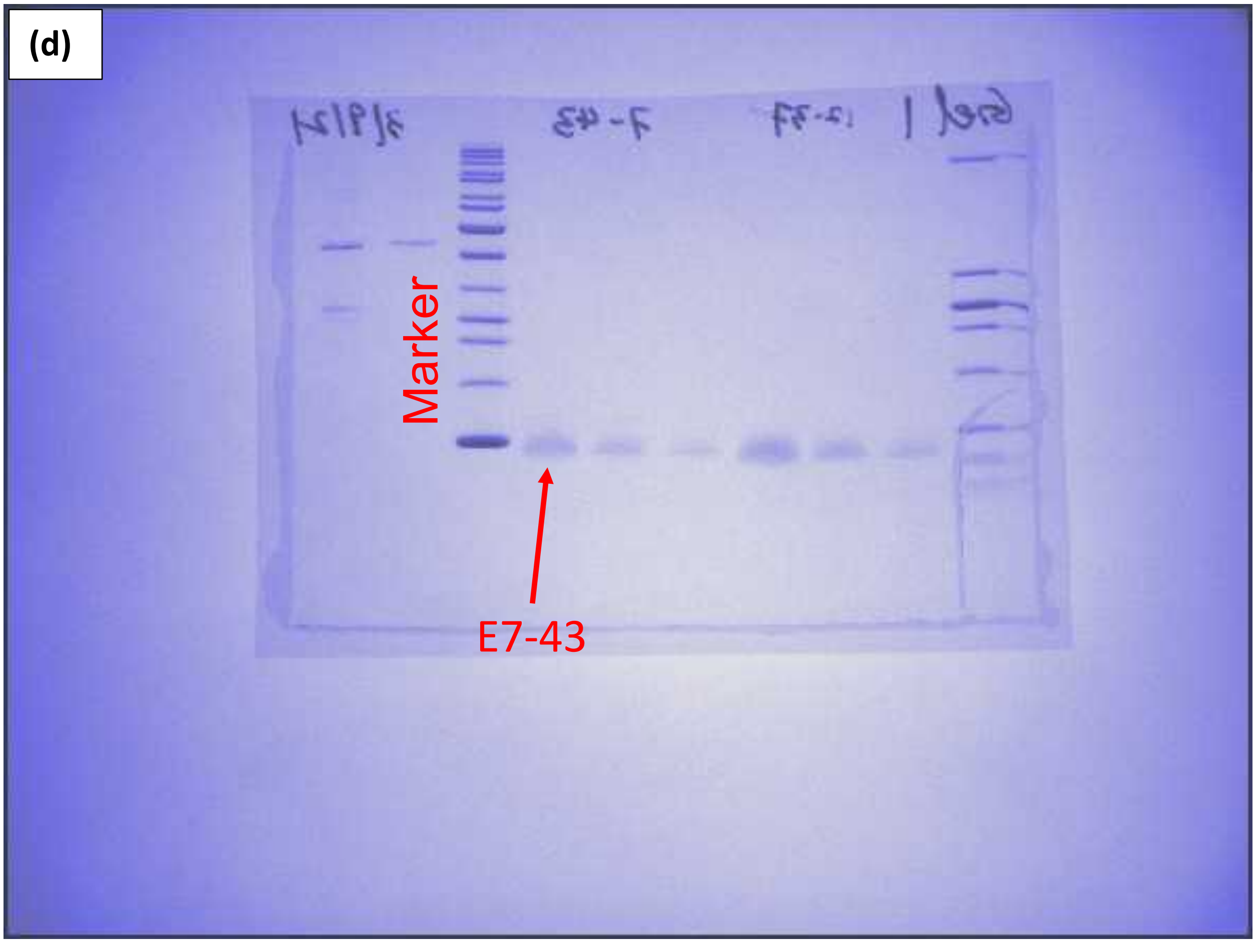

Supplementary Figure 10. Uncropped gel images. (d) $E_{7\text{-}43}$; cropped version shown in Figure 1d.

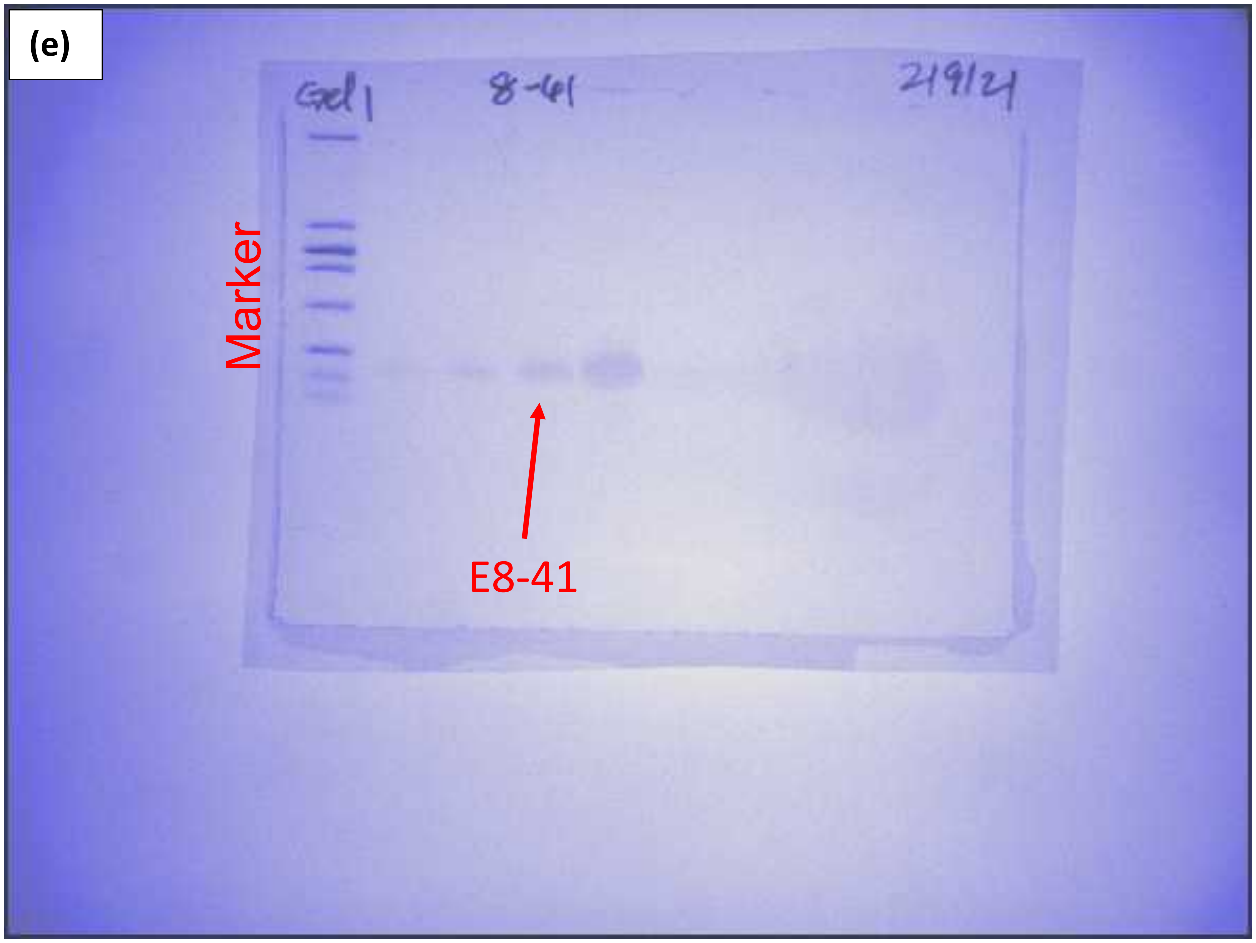

Supplementary Figure 10. Uncropped gel images. (e) E_{8-41}; cropped version shown in Figure 1e, with the aspect ratio increased by 33%.

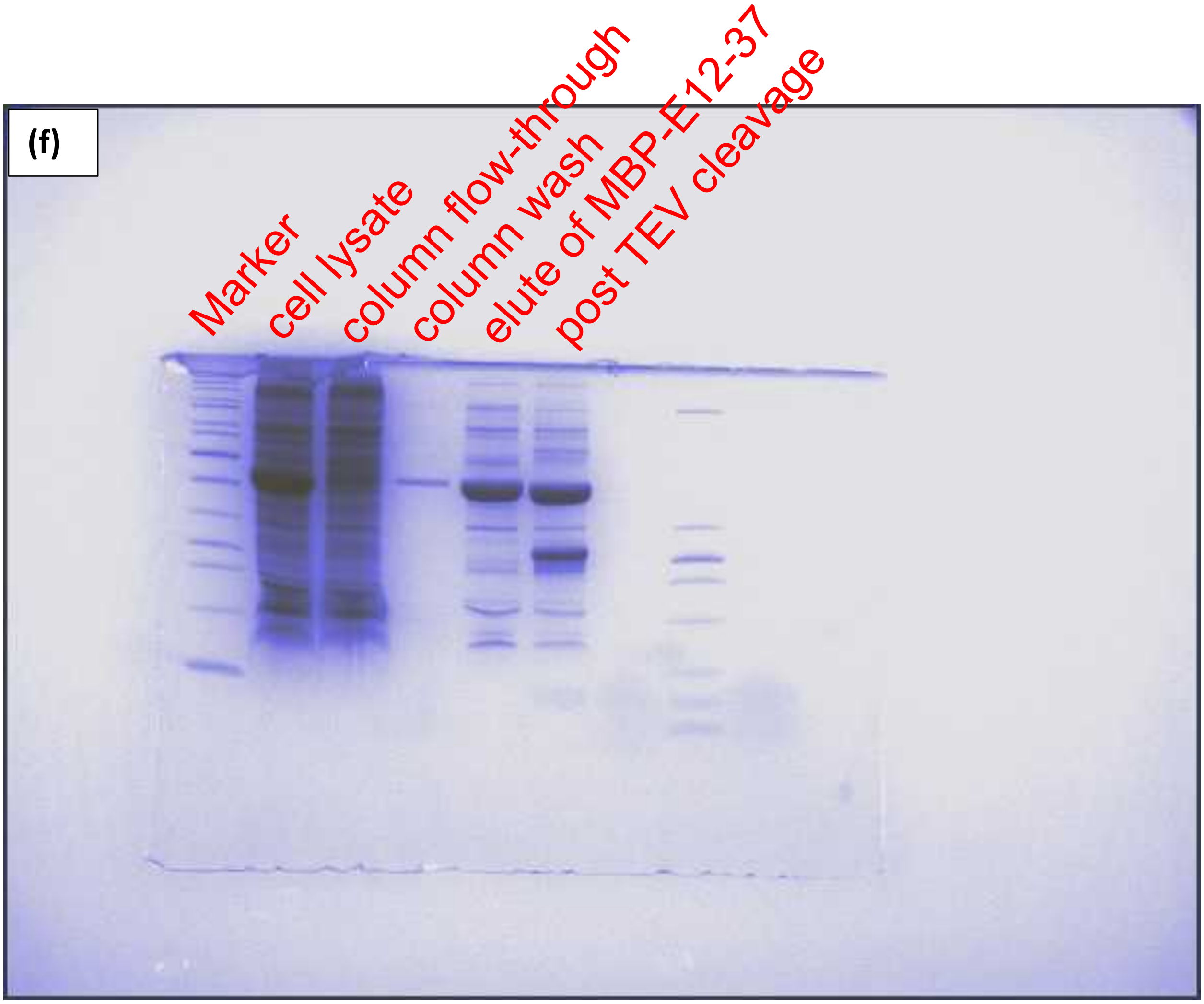

Supplementary Figure 10. Uncropped gel images. (f) $E_{12\text{-}37}$ purification; cropped version shown in Supplementary Figure 2, with the aspect ratio decreased by 8%.
